# Inhibition of glutamate oxaloacetate transaminase 1 in cancer cell lines results in altered metabolism with increased dependency of glucose

**DOI:** 10.1186/s12885-018-4443-1

**Published:** 2018-05-11

**Authors:** Xiaoshan Zhou, Sophie Curbo, Fuqiang Li, Shuba Krishnan, Anna Karlsson

**Affiliations:** 1 Division of Clinical Microbiology, Department of Laboratory Medicine, Karolinska Institute, Karolinska University Hospital, 141 86 Stockholm, Sweden; 2 BGI-Shenzhen, Beishan Industrial Zone, 518083 Yantian District, Shenzhen, China

**Keywords:** Glutamate oxaloacetate transaminase 1, Glucose, Cancer, Glycolysis, Redox regulation, Metabolic regulation

## Abstract

**Background:**

Glutamate oxaloacetate transaminase 1 (GOT1) regulates cellular metabolism through coordinating the utilization of carbohydrates and amino acids to meet nutrient requirements. KRAS mutated cancer cells were recently shown to rely on GOT1 to support long-term cell proliferation. The aim of the present study was to address the role of GOT1 in the metabolic adaption of cancer cells.

**Methods:**

GOT1-null and knockdown cell lines were established through CRISPR/Cas9 and shRNA techniques. The growth properties, colony formation ability, autophagy and selected gene expression profiles were analysed. Glucose deprivation decreased the viability of the GOT1-null cells and rescue experiments were conducted with selected intermediates. The redox NADH/NAD^+^ homeostasis as well as lactate secretion were determined. GOT1 expression levels and correlation with survival rates were analysed in selected tumor databases.

**Results:**

Inhibition of GOT1 sensitized the cancer cells to glucose deprivation, which was partially counteracted by oxaloacetate and phosphoenol pyruvate, metabolic intermediates downstream of GOT1. Moreover, GOT1-null cells accumulated NADH and displayed a decreased ratio of NADH/NAD^+^ with nutrient depletion. The relevance of GOT1 as a potential target in cancer therapy was supported by a lung adenocarcinoma RNA-seq data set as well as the GEO:GSE database of metastatic melanoma where GOT1 expression was increased. High levels of GOT1 were further linked to poor survival as analysed by the GEPIA web tool, in thyroid and breast carcinoma and in lung adenocarcinoma.

**Conclusions:**

Our study suggests an important role of GOT1 to coordinate the glycolytic and the oxidative phosphorylation pathways in KRAS mutated cancer cells. GOT1 is crucial to provide oxaloacetate at low glucose levels, likely to maintain the redox homeostasis. Our data suggest GOT1 as a possible target in cancer therapy.

**Electronic supplementary material:**

The online version of this article (10.1186/s12885-018-4443-1) contains supplementary material, which is available to authorized users.

## Background

The metabolic reprogramming of cancer cells is likely a tool for the cells to rapidly respond to micro-environmental changes and to the demand of nutrients for cell growth and survival [1–4]. Evidence shows that cancer cells with a KRAS mutation or an electron transport chain defect depended on glutamate oxaloacetate transaminase 1 **(**GOT1) to support cell proliferation [5–7]. GOT1 reversibly catalyzes the interconversion of aspartate and oxaloacetate (OAA), and thus coordinates the carbohydrate and amino acid metabolism. In pancreatic ductal adenocarcinoma (PDAC) cells, glutamine-derived aspartate was shown to be converted to OAA by GOT1 and GOT1 was also involved in the maintenance of the redox homeostasis through the sequential conversion of OAA into pyruvate. This reprogramming of glutamine metabolism was driven by KRAS, one of the most common genetic alterations in pancreatic cancer [6]. However, the metabolic KRAS-GOT1 link was not found in primary human pancreatic tumors and the mechanisms behind the GOT1 involvement have remained unclear [8]. Interestingly, in contrast to PDAC cells, where aspartate was converted to OAA by GOT1, cancer cells with defects in the electron transport chain reversibly used GOT1 to provide aspartate from OAA to maintain cell growth [5]. Through further analysis of GOT1 with the Oncomine database, we found the GOT1 gene expression levels to vary in different types of cancers, for instance the GOT1 expression was up-regulated in breast and lung cancer [9–12], while down-regulated in brain and colorectal cancer [13–16]. Taken together, these data do not only show the importance of GOT1 in supporting cancer cell proliferation, but also imply a complexity of the GOT1 function in cancer cell metabolism. The microenvironment and nutrient levels in solid tumors are constantly changed [17]. To survive such conditions, cancer cells have to rapidly alter metabolic pathways. We hypothesized that GOT1 can be used by tumor cells to deal with unfavorable growth conditions. Therefore, studies of GOT1 may give new insights in cancer cell metabolism, and may also provide novel targets for cancer treatment. Here, we found that GOT1 is involved in the regulation of glycolytic metabolism and redox homeostasis. Inhibition of GOT1 leads to increased glucose consumption and lactate secretion rates. Moreover, GOT1-null 143B osteosarcoma cells showed accumulation of NADH and decreased NADH/NAD^+^ ratios when exposed to nutrient depletion. Our results also show that the GOT1 pathway is dispensable for cancer cells when nutrients are sufficient. However, GOT1 is indispensable for cell survival at low nutrient levels, probably as the key source of OAA to fuel the rewired metabolic pathways.

## Methods

### Cell lines, plasmids and reagents

143B (ATCC® CRL-8303™) and A549 (ATCC® CCL-185™) cells were cultured in DMEM supplemented with 10% FBS, 1% penicillin and streptomycin at 37 °C, 5% CO_2_. siRNA expression vector pSilencer™ Puro Expression Vectors kit (Applied Biosystems,Life technologies), the CRISPR/CAS 9 plasmid pSpCas9(BB)-2A-GFP was from Addgene and TurboFect transfection reagent from Thermo Scientific. The kits: The quick ligation kit (NEB); RNeasy mini kit (Qiagen); High Capacity cDNA Reverse Transcription Kit (Applied Biosystems); KAPA SYBR^®^ FAST qPCR Kit (Kapa Biosystems); Cell Proliferation Kit II (XTT) (Sigma-Aldrich); Reagents: L-glutamine solution, dialyzed FBS, glucose, galactose, glycine, serine, L-aspartic acid sodium salt monohydrate, OAA, phosphoenolpyruvate (PEP), β-nicotinamide adenine dinucleotide sodium salt and aminooxyacetate (AOA), Antimycin A and 2-thenoyltrifluoroacetone were from Sigma. The glucose and pyruvate-free and glutamine free-DMEM were from Thermo Fisher Scientific.

### Establishment of GOT1 knockout cell line with CRISPR/Cas 9 system

The targeting sequences for single strand guide RNAs were determined with the online tool CRISPR RNA Configurator from Dharmacon, and the sequence of AGTCTTTGCCGAGGTTCCGC was selected for making CRISPR/Cas 9 construct. Briefly, sgRNA specifying oligos (5′---caccgAGTCTTTGCCGAGGTTCCGC---3′; 5′---aaacGCGGAACCTCGGCAAAGACTc---3′) were synthesized, annealed and cloned into pSpCas9(BB)-2A-GFP vector as described by Ran FA [18]. The plasmid with specific insertion was confirmed by DNA sequencing and purified for cell transfection. The 143B cell line was used for cell transfection. The cell transfection experiment was performed according to the manufacturer’s instructions. After 48 h post-transfection, the single GFP expressing cells were sorted and seeded in 96-well plates with BD FACSAria™ III. The clones with indel mutations were pre-screened by real-time quantitative PCR with two pairs of primers. Primers specific for wild allele: Forward 5’-AGTCTTTGCCGAGGTTCCG-3′; Reverse 5’-GTGCGATATGCTCCCACTC-3. Primers for wild and mutated alleles: Forward 5′- TGCTCCTGAGTTCTCCATTG-3′; Reverse 5′- AACAGAAACCGGTGCTTCAT-3′. For mutated allele primers, the forward primer was located in the region where expected indel might occur. If the indel mutation is introduced via CRISPR7CAS9 system, the efficiency of the PCR will be compromised. After the pre-screening, the clones with lower GOT1 mRNA level were further confirmed by gene sequencing. Primers for amplification of the DNA fragment of the mutated DNA region for sequencing were: Forward: 5’-GCTAATAGCGTTCCTTCTCCCC-3′; Reverse: 5’-TACATCCTTACCTCCCACTCCC-3′. The knockout of GOT1 was further confirmed with Western blot with specific anti-GOT1 antibody (Abcam). For loading control, the primary antibody was β-actin (Sigma). The secondary antibody was anti-mouse (Santa Cruz).

### Establishment of stable GOT1 knock-down cell lines in 143B and A549 cells with siRNA

GOT1 specific siRNAs and mock control siRNA expression plasmids were prepared as described by the manufacturer. Briefly, two specific oligonucleotides were designed using online tools BLOCK-iT™ RNAi designer (http://rnaidesigner.lifetechnologies.com/rnaiexpress/). The siRNA target sequences are 5′---TAGCCTAAATCACGAGTAT---3′ and 5′---TGGACAGGTAATGTGAAGA---3′ respectively. The two complimentary oligonucleotides were annealed and ligated into pSilencer 2.1-U6 puro siRNA expression vector and transfected into 143B and A549 cells with TurboFect Transfection Reagent. Stable expression cells were selected with puromycin at 1000 ng/ml for 2 weeks and the knock-down clones were screened with real-time PCR with the primers used for the CRISP/Cas 9 mutated cells. The GOT1 knock-down cell lines and mock controls were maintained with puromycin at concentration of 200 ng/ml.

### Growth curves of CRISPR/cas9 knockout and siRNA knock-down 143B cell lines

25 × 10^3^ cells in exponential phase were seeded in 24-well plates in triplicate. The cell numbers were counted every day for 5 days with a Bürker chamber under light microscope.

### Soft agar colony assay

The soft agar experiment was performed as described previously [19]. Briefly, 500 CRISPR/Cas9 mutated and wild type143B cells were suspended in 0.35% agarose in DMEM supplemented with 10% FBS, 1% penicillin and streptomycin, and seeded over a basal layer of 0.5% agarose. For 143B siRNA knock-down cell lines, 500 cells were used for the soft agar assay. For A549 cell lines, 1000 cells were seeded in the plates. After 10 days culture for 143B cell lines and 3 weeks for A549 cell lines at 37 °C, 5% CO_2_, plates were stained with 0.01% crystal violet for 1 h, and colonies were scored manually from 3 wells for each cell type.

### AOA treatment

Briefly, wild type 143B and A549 cells were seeded in triplicate in 96-well plates at density of 1 × 10^4^ cells per well. After 24 h, the AOA was added into the wells at different concentrations as indicated in the figures. The cell viability was measured with XTT assay 24 h after the addition of AOA.

### Gene expression profile changes determined with real-time PCR

Since GOT1 has been linked to growth arrest, metabolism and oxidative stress, genes involved in these pathways were analysed with real-time PCR. The gene expression levels were analysed for*CDNK1A, HIF1α, ATG5, BECN1, BIP, G6PC3, CHOP, GADD34* and *GOT1*. *S18* was used as a loading control. Briefly, 2 × 10^5^ wild type and mutated 143B cells were seeded in 6-well plates in triplicate. After 24 h, three wells of each wild type and mutated 143B the cells were collected for total RNA extraction as time point zero. For the rest of the wells, the medium was replaced with glucose-free medium. After 4 h incubation, the total RNA was extracted and the cDNA was synthesized according to the manufacture’s instruction. The real-time PCR experiments were conducted using an Applied Biosystems 7500 Fast Real-time PCR with the following primers:

*P21*: 5’-CAGACCAGCATGACAGATTTC-3′; 5’-TTAGGGCTTCCTCTTGGAGA-3’.

*HIF1α*: 5’-TGCAACATGGAAGGTATTGC-3; 5’AATGGGTTCACAAATCAGCA-3′.

*ATG5*: 5’-TGGGATTGCAAAATGACAGA -3′; 5’-TTTCCCCATCTTCAGGATCA -3’.

*BENC1*:5′- CCAGGATGGTGTCTCTCGCA -3′; 5′- CTGCGTCTGGGCATAACGCA-3’.

*BIP*: 5’-CTCAACATGGATCTGTTCCG -3′; 5’-CCAGTTGCTGAATCTTTGGA -3’.

*G6PC-3*: 5’-ATAATGACGGCCCTGTCTTC-3′; 5’-TGGTGAGGGAAATGTGCTAA-3’.

*CHOP*: 5’-TCATACATCACCACACCTGA-3′; 5’-TAGGTACCCCCATTTTCATC-3′.

*GADD34*: 5’-ATGATGATGGCATGTATGGT-3′;'5’-TTAACTCCCTCCTCTTCAGC-3’.

*S18*: 5’-TCACTGAGGATGAGGTGGAA-3′; 5′- GCTTGTTGTCCAGACCATTG -3′.

### Analysis of autophagy with western blot

Wild type and GOT1-null 143B cells were grown at a density of 1 × 10^6^ in 10 ml DMEM with 10% FBS, 1% % penicillin and streptomycin into two 10 cm Petri dishes for each cell types. After 24 h, one plate from each cell type was collected. The medium in the rest of plates was replaced with glucose-free DMEM supplemented with 10% dialyzed FBS, 1% penicillin and streptomycin, and the cells were cultured for 4 h, then harvested. The cells were lysed in RIPA buffer, and 25 μg total protein was used for Western blot. To study autophagy in GOT1-null cells, the primary antibody was anti-LC3 (Sigma, L8918), and the secondary antibody was donkey anti-rabbit (Santa Cruz). For loading control, the primary antibody was β-actin (Sigma). The secondary antibody was anti-mouse (Santa Cruz).

### Glucose and glutamine deprivation

To investigate the glucose and glutamine dependency of GOT1-null 143B cells, wild type and GOT1-null 143B cells were seeded in 96-well plates in triplicate at a density of 5 × 10^3^ cells per well in DMEM supplemented with 10% FBS, 1% penicillin and streptomycin. After 24 h, the medium was replaced with DMEM with glucose concentration at 0, 0.2, 1 and 4.5 g/L glucose. For glutamine deprivation, the cells were grown in DMEM with or without glutamine at concentration of 2 mM. The 10% dialyzed FBS was added into the medium. After 24 h, the cell viability was determined with XTT assay.

### Metabolites supplement experiment

To study the rescue of GOT1-null 143B cells with different metabolites, 5 × 10^3^ wild type and mutated 143B cells were seeded in 96-well plates in triplicate. After 24 h, the cell medium was replaced with glucose free DMEM supplemented with different metabolites: galactose, glycine, serine, pyruvate, aspartate, OAA and PEP. The concentrations of the metabolites are indicated in the figures. For GOT1 siRNA knock-down A549 cells, the same number of non-template control (NTC) cells and siRNA-1 cells were seeded in 96-well plates in triplicate. After 24 h, the culture was replaced with DMEM supplemented with different metabolites as indicated in the figures. For the combined AOA and metabolites experiments, 5 × 10^3^ wild type 143B and A549 cells were seeded in 96-well plates, and after 24 h the medium was replaced with glucose-free DMEM supplemented with AOA and metabolites as indicated in the figures. Dialyzed FBS was used in these experiments. The cell viability was determined with XTT assay.

### H_2_O_2_ challenge

For H_2_O_2_ resistance, both wild type and GOT1-null 143B cells were seeded in 96-well plates in triplicate at density of 1 × 10^4^. After 24 h, the medium was replaced with fresh DMEM with H_2_O_2_ concentrations at 0.2, 2 and 20 mM. The viability was determined with XTT after 24 h.

### Nutrient depletion and rescue of GOT1-null 143B cells with different metabolites

To deplete nutrients in the medium, both wild type and GOT1-null 143B cells were seeded in 96-well plates in triplicate at a density of 5 × 10^3^. The cells were continuously grown for 4 days without changing medium. Then the medium was replaced with glucose-free and 1 g/L glucose DMEM supplemented with 10 mM aspartate, 5 mM OAA, 2.5 mM PEP respectively. For glucose-free DMEM, malate (10 mM), succinate (10 mM) and NAD^+^ (100 μM) were also tested. The cell morphological changes were recorded. The cell viability treated with malate, succinate and NAD^+^ for 24 h were determined with XTT assy.

### Antimycin a and 2-thenoyltrifluoroacetone treatment

Both wild type and GOT1-null 143B cells were seeded in 96-well plates in triplicate at density of 5 × 10^4^. After 24 h, 0.1 μM of antimycin A and 1 mM of 2-thenoyltrifluoroacetone (TTFA) were added into the medium respectively. The viability of cells was measured after 48 h with XTT assay.

### Cell viability determination

Cell viability was determined with XTT assay. Briefly, 50 μl of XTT labelling reagent and 1 μl of electron coupling reagent were mixed and 50 μl of mixture added into each well of a microtiter plate. The plate was incubated in a humidified atmosphere for 3 h and the absorbance was measured using an ELISA reader (Infinite® M200, Tecan trading AG) at 450 nm with a reference wavelength at 690 nm. All the data were normalized with untreated control groups in the experiments.

### Measurements of the levels of glucose, lactate and NADH/NAD^+^

Wild type and GOT1-null 143B cells were seeded in 24-well plates in triplicate at density of 5 × 10^4^. The culture supernatants were collected at day 2 and day 4. The concentrations of glucose and lactate in the supernatants were measured with Glucose assay kit (Biovision) and Lactate assay kit (Sigma) respectively. The NADH/NAD^+^ levels were analysed with NADH/NAD^+^ assay kit from Sigma. For NADH/NAD^+^ levels in nutrient-complete condition, 1 × 10^6^ wild type and mutated 143B cells were seeded in 10 cm Petri dishes, after 24 h the cells were harvested; For the NADH/NAD^+^ levels in low nutrient condition, the cells were continuously cultured for 4 days and then harvested. The assays were performed according to the instruction of manufacture.

### Metformin treatment

Wild type A549, 143B and Mia PaCa-2 cells were seeded in 96-well plates in duplicate at a density of 5 × 10^3^. After 24 h, the medium was replaced with complete, glucose-free and glutamine-free DMEM with metformin at a concentration of 5 mM. The cell viability was measured 24 h later with XTT.

### Data set analysis and mining

To confirm the relevance of GOT1 to cancer growth in vivo, we first analyzed the GOT1 expression profile in a lung cancer RNA-seq data set [20]. In order to confirm the analysis result from our own data set, we also mined and examined another two data sets: the early stage NSCLC data base GEO:GSE19188 [21] and the metastatic melanoma data base GEO: GDS3966 [22]. GEPIA is a newly established gene expression analysis web server [23], which integrates TCGA and GTEx databases, totally including 9736 cancers and 8587 normal samples, and improves the efficiency in various differential analyses. With the GEPIA web tool, we investigated the GOT1 expression patterns and the relationship between GOT1 expression profiles and the overall survival rates. The GOT1 gene expressions were checked in 33 types of cancers, and the overall survival rates were also investigated with quartile cut-off.

### Statistics

Unpaired student’s t-test, one way ANOVA or Wilcoxon test was used for statistics analysis.

## Results

### Down -regulation of GOT1 inhibits cancer cell growth and migration

**Fig. 1:**
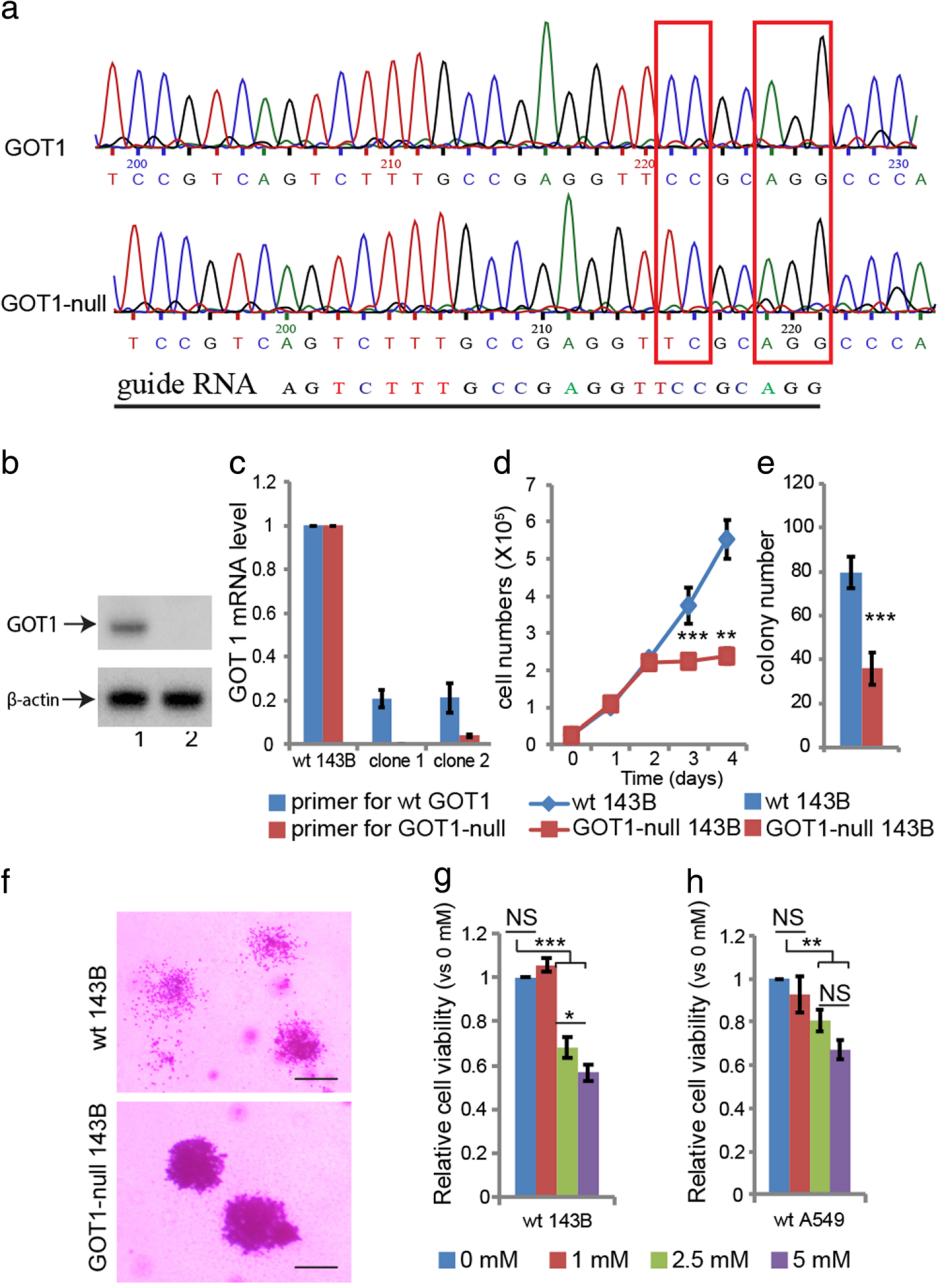
Characterization of GOT1-null cells. **a** A single base pair CG deletion in exon 1 of GOT1 introduced via CRISPR/Cas9 in 143B cells. **b** Western-blot of GOT1. 1: wild type 143B; 2: mutated GOT1. **c** Expression levels of wild type and GOT1-null mRNA. **d** Growth curves of wild type and GOT1-null 143B cells. **e** Colony formation of GOT1-null 143B cells in soft agar. **f** Decreased migration of GOT1-null 143B cells. Bars indicate 50 μm. **g** AOA treatment in wild type 143B. **h** AOA treatment in wild type A549 cells. All the experiments have been repeated 3 times, and data are represented as mean ± s.d. Unpaired student’s t-test was performed for **d**, **e**. One-way ANOVA was performed for **g** and **h**. *** *p* < 0.001, ** *p* < 0.01, * *p* < 0.05; NS: not significant

We established a GOT1-null cell model using the CRISPR/CAS9 gene editing technique [18]. A deletion was introduced into exon 1 of GOT1 in the 143B osteosarcoma cell line, which led to a reading frame-shift mutation (Fig. 1a). The total GOT1 knockout was confirmed with Western blot (Fig. 1b). The mutation also decreased the stability of the GOT1 mRNA with a transcript level almost undetectable as compared to the wild type control cells (Fig. 1c). Cells with mutated GOT1 showed slower growth, formation of fewer colonies in soft agar and impaired cell migration in soft agar as compared to wild type cells (Fig. 1d-f). The growth inhibitory effect was confirmed with aminooxyacetate (AOA) [6], a GOT1 inhibitor (Fig1g and h) and in siRNA knockdown of GOT1 in 143B and A549 cell lines (Additional file 1: Figure S1a-f).

### Disruption of GOT1 leads to increased glucose dependency

**Fig. 2:**
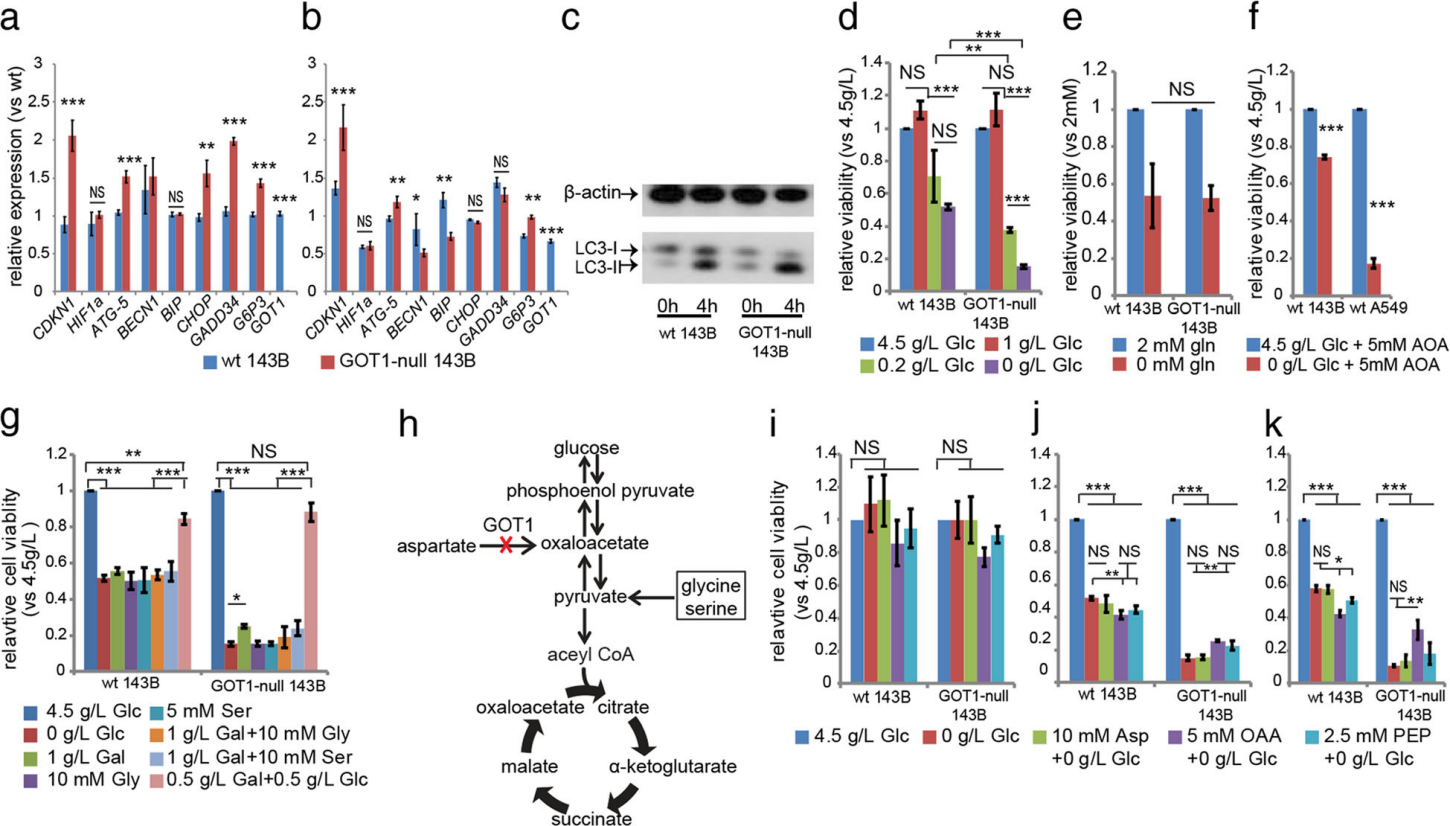
Gene expression profile and rescue of GOT1-null 143B cells with different metabolites. **a** Gene expression profile before glucose deprivation **b**, Gene expression profile 4 h after glucose deprivation. **c** Changes of LC3-II levels before and after glucose deprivation for 4 h. **d** Cell viability in different concentrations of glucose for 24 h. **e** Cell viability upon glutamine deprivation for 24 h. **f** Relative viabilities of wild type 143B and A549 cells in medium with glucose concentration at 4.5 g/L or 0 g/L in the presence of AOA at concentration of 5 mM. **g** Relative cell viability in medium supplemented with different metabolites (Glc: glucose; Gly: glycine; Ser: serine; Gal: galactose). **h** Illustration of intermediates in gluconeogenesis pathway. **i** Wild type and GOT1-null 143B cells grown in glucose free medium supplemented with 10 mM aspartate (Asp), 5 mM OAA, or 2.5 mM PEP for 4 h, **j**, 16 h and **k,** 24 h. All the experiments have been repeated three times, and data are represented as mean ± s.d. One-way ANOVA test was performed for **d**, **g**, **i**, **j** and **k**. Unpaired student’s t-test was performed for **a**, **b**, **e** and **f**. *** *p* < 0.001;** *p* < 0.01; * *p* < 0.05; NS: not significant

To explore the underlying mechanisms, a gene expression profile was performed to determine genes affected by the GOT1disruption. 6 groups of genes were included: the hypoxia-related gene *HIF-1α*, the cell cycle-related *p21* gene, the autophagy-related genes (*ATG5* and *BECLIN1*), the endoplasm-reticulum-stress-related genes (*CHOP* and *GADD34*), the glucose-controlling gene *BIP* and the gluconeogenesis-pathway-gene glucose-6-phosphatase 3 (*G6PC3*). The expressions of *p21*, *ATG5* and *G6PC3* were up-regulated both during normal growth conditions and after glucose deprivation. The levels of *CHOP*, *GADD34*, *BECLIN1* and *BIP* decreased after glucose deprivation (Fig. 2a and b). Western blot analysis showed that inhibition of GOT1 led to signs of autophagy with increased ratios between the activated and inactivated forms of the autophagosome protein LC3A upon glucose deprivation (Fig. 2c). The viability of the GOT1-null cells highly relied on the extracellular glucose concentration, whereas deprivation of glutamine had no effect (Fig. 2d and e). Wild type cells treated with the GOT1 inhibitor AOA also increased their glucose dependency (Fig. 2f). These results indicated that GOT1 was involved in glucose metabolism and cell stress regulation.

### OAA and PEP, but not aspartate, were able to improve the viability of the GOT1-null cells upon glucose deprivation

To further investigate the biological pathways involved in the observed metabolic changes in the GOT1-null cells, rescue experiments were performed with different metabolites. The addition of galactose only partially rescued the viability of GOT1-null cells during glucose deprivation and neither serine nor glycine was able to rescue the cells. The only additive that did rescue cell viability was a low concentration of glucose (Fig. 2g). We subsequently grew the cells in the absence of glucose and added aspartate, OAA or PEP. These intermediates are substrates in the gluconeogenesis pathway as shown in Fig. 2h. The cell viability was not significantly changed after 4 h (Fig. 2i). After 12 h the cell viability was impaired in both wild type and GOT1-null 143B cells although the GOT1-null cells displayed a more pronounced loss of viability (Fig. 2j). In contrast to the wild type cells, addition of OAA or PEP improved the viability of the GOT1-null cells after 12 h and 24 h (Fig. 2j-k). Addition of aspartate did not improve the viability of the cells at any time point.

The results were confirmed in wild type 143B and A549 cells treated with AOA. Supplement of OAA into glucose-free medium was able to maintain the cell viability (Additional file 2: Figure S2a-d) and the addition of OAA also partially rescued two GOT1 siRNA knock-down cell lines constructed from two different siRNA sequences (Additional file 2: Figure S2e and f). Aspartate did not improve the cell viability in neither wild type cells nor siRNA knock-down cells (Additional file 2: Figure S2a-f). Due to the acidity of OAA, increasing OAA concentration further than 10 mM failed to improve the viability of GOT1-null 143B cells. However, the rescue ability of OAA was much higher in GOT1-null 143B cells than in wild type 143B cell (Additional file 2: Figure S2 g). These results indicated that GOT1 supplies OAA and subsequently PEP to the gluconeogenesis pathway to maintain viability during nutrient crisis.

### GOT1 is an important source of OAA for metabolic adaptation of cancer cells to nutrient level changes

**Fig. 3:**
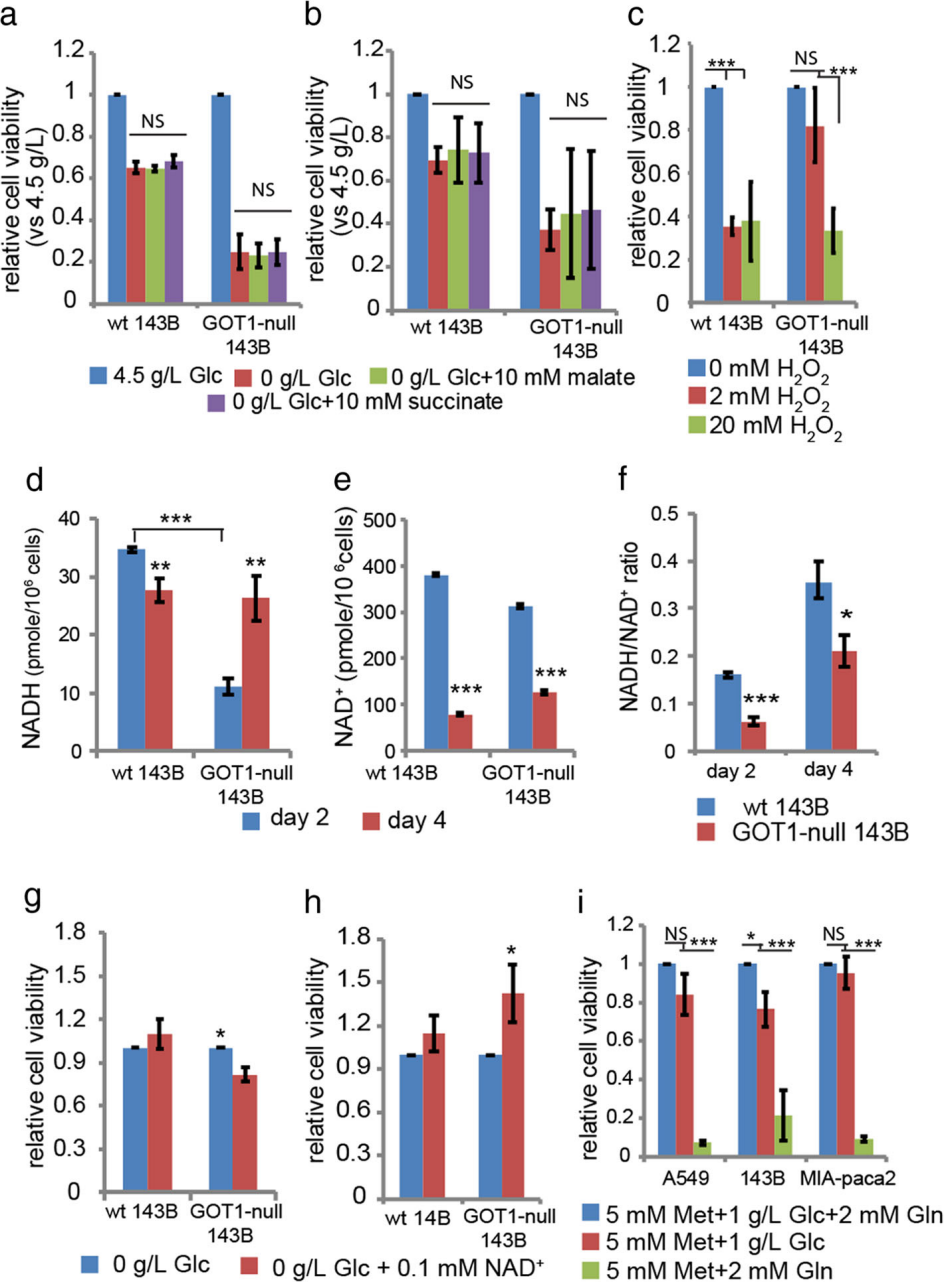
Redox homeostasis and NADH/NAD^+^ ratio in GOT1-null 143B cells. Cell viability compared to cells grown in 4.5 g/L glucose in **a**, dividing cells or **b**, confluent cells 24 h after treatment with fresh medium without glucose, and supplemented with 10 mM malate or 10 mM succinate for 24 h. **c**, Response to H_2_O_2_ exposure. Cells were treated with H_2_O_2_ at concentrations of 0, 2 and 20 mM, respectively. The cell viability was determined 24 h after treatment. The levels of **d**, NADH and **e**, NAD^+^ and **f**, the ratios of NADH to NAD^+^, after 2 days (sufficient nutrition medium) and 4 days (nutrition-depleted medium) of cell growth. **g**, Cell viability after 24 h of 0.1 mM of NAD^+^ treatment at day 2. **h**, Cell viability after 24 h of 2.5 mM of NAD^+^ treatment at day 4. **i**, Relative cell viability in wild type A549, 143B and Mia-paca-2 cells treated with metformin (Met) in indicated culture medium for 24 h. All the experiments have been repeated at least 3 times independently. Data are presented as mean ± s.d. from 3 independent experiments. One-way ANOVA analysis was performed for **a**, **b**, **c** and **i**. Unpaired student’s t-test was performed for **d-h**. *** *p* < 0.001;** *p* < 0.01;* *p* < 0.05; NS: not significant

In addition to GOT1, mammalian cells can produce OAA in the cytosol through malate dehydrogenases and ATP citrate lyase. To further delineate the contributions from these pathways, the GOT1-null cells were cultured in glucose-free medium supplemented with malate and succinate, respectively. Theoretically, the addition of malate or succinate should provide citrate via the TCA cycle, assuming that the mitochondrial TCA function is not impaired due to GOT1 inhibition. Based on the results of impaired growth of the GOT1 inhibited cells after 3 days in culture (Fig. 1d and Additional file 1: Figure S1b), we tested the rescue ability of these metabolites at day 1, representing dividing cells, and day 4, representing confluent cells. The addition of malate and succinate were not able to rescue the GOT1-null cells upon glucose deprivation neither at day 1 (Fig. 3a) nor at day 4 (Fig. 3b). These results indicated that GOT1 is a major source of OAA that can be shunted into rewired metabolic pathways in order to maintain cell viability when glucose levels are low.

### Inhibition of GOT1 causes NADH accumulation upon chronical nutrient depletion

The GOT1-null cells were more resistant to 2 mM H_2_O_2_ than the wild type 143B cells, which indicated a shift in the redox balance with an increased capacity of the GOT1-null cells to accept the electrons provided from H_2_O_2_ (Fig. 3c). To determine the redox state of the cells we analysed the levels of NADH and NAD^+^ and found that the NADH level was significantly lower in the GOT1-null cells than in the wild type cells when cultured in medium with high levels of nutrients, which is consistent with the results of the H_2_O_2_ challenge experiments. However, after 4 days of cell growth with a concomitant decrease of nutrients in the cell medium the NADH level increased in the GOT1-null cells whereas it decreased in the wild type cells (Fig. 3d). Although the NAD^+^ levels decreased in both the GOT1-null and the wild type cells (Fig. 3e), the ratios of NADH/NAD^+^ were significantly lower in the GOT1-null cells as compared to the wild type cells cultured for 2 days (nutrient-enough medium) and cultured for 4 days (nutrient-depleted medium) (Fig. 3f). It has been shown that extracellular application of NAD was able to restore intracellular NAD(P) levels, and NAD uptake may play a role in physiological NAD homeostasis [24]. Therefore, This new steady state redox homeostasis was further tested with NAD^+^ treatment. When exposing GOT1-null cells that had been growing under nutrient-enough conditions to glucose-free medium with the addition of NAD^+^ the viability decreased (Fig. 3g), while the viability of the GOT1-null cells grown under chronic nutrient-depleted stress increased upon NAD^+^ treatment (Fig. 3h). In addition, only NAD^+^ was able to partially prevent cell morphological changes upon replacing the nutrient-depleted medium with fresh medium without glucose (Additional file 3: Figure S3). The NADH level changed in the GOT1-null 143B cells similarly to cells treated with metformin, a well-known inhibitor of gluconeogenesis [25, 26]. Consistent with the increased glucose dependency in the GOT1-null 143B cells, treatment of different cancer cell lines with metformin also increased glucose dependency similarly to GOT1-null 143B cells (Fig. 3i).

### The increased glucose consumption is due to increased lactate secretion

**Fig. 4:**
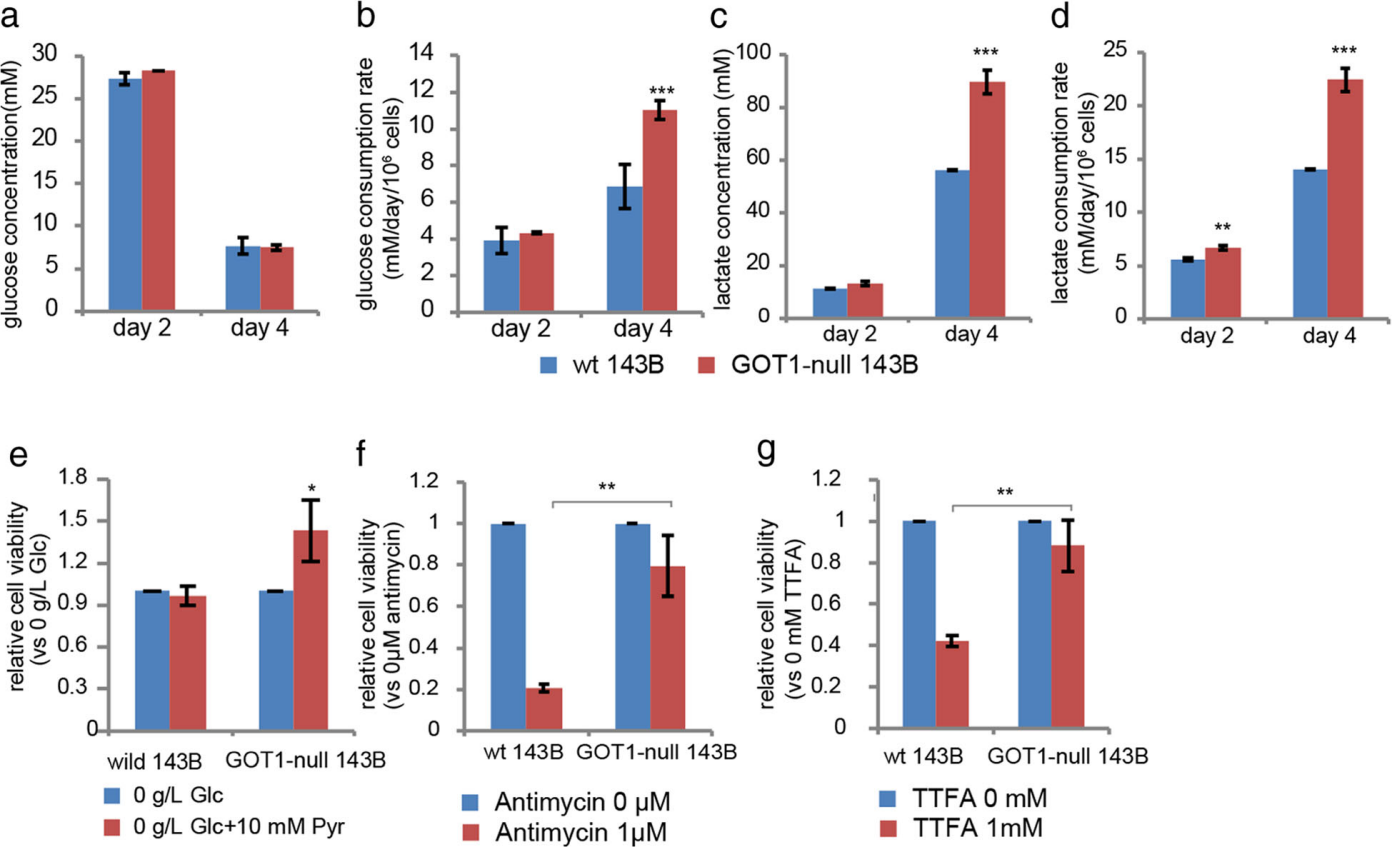
Glucose consumption and lactate secretion rates and response to mitochondrial complexes inhibitors in GOT1-null 143B cells. **a** Glucose concentrations, **b** Glucose consumption rates, **c** Lactate concentrations, **d** Lactate secretion rates determined in culture supernatants after 2 days (dividing cells) and 4 days (confluent cells). Cell viability in cells grown in complete medium for 2 days and then grown for 24 h in medium **e** without glucose only or supplemented with 10 mM pyruvate (Pyr), **f** with 0 or 1 μM antimycin A, **g** with 0 or 1 mM (TTFA). All experiments have been repeated 3 times independently. Data are presented as mean ± s.d. Unpaired student’s t-test was performed. *** *p* < 0.001; ** *p* < 0.01; * *p* < 0.05

To investigate the mechanism underlying increased glucose dependency in the GOT1-null cells, we first measured the glucose consumption rate and lactate levels in culture medium. The glucose levels in the supernatants were not significantly changed at day 2 and day 4 (Fig. 4a). Considering the different growth curves between wild type and GOT1-null cells (Fig. 1c and Additional file 1: Figure S1b), we normalized the values with cell numbers. The glucose consumption rate was significantly higher in the GOT1-null cells than the wild type control cells (Fig. 4b). Cancer cells secret lactate at high levels, and we therefore investigated the lactate levels in the culture supernatants [27]. The level of lactate was significantly increased in the supernatant at day 4 (Fig. 4c). The lactate secretion rate started to increase at day 2, and was higher at day 4 (Fig. 4d). To investigate if pyruvate was able to improve the viability of GOT1-null 143B cells, we replaced the old medium with glucose-free medium supplemented with pyruvate at day 2. As seen in Fig. 4e pyruvate significantly increased the cell viability upon glucose deprivation after 24 h treatment. In addition, the viability of GOT1-null cells was not significantly affected by treatment with TTFA and antimycin A, inhibitors of mitochondrial complex II and III respectively, as show in Fig. 4f and g [28, 29]. Altogether, these data indicated that the increased glucose consumption was related to increased aerobic glycolysis and excretion of lactate into the extra-cellular environment.

### Disrupted GOT1 induced rapid ischemic-like cell death upon glucose deprivation

**Fig. 5:**
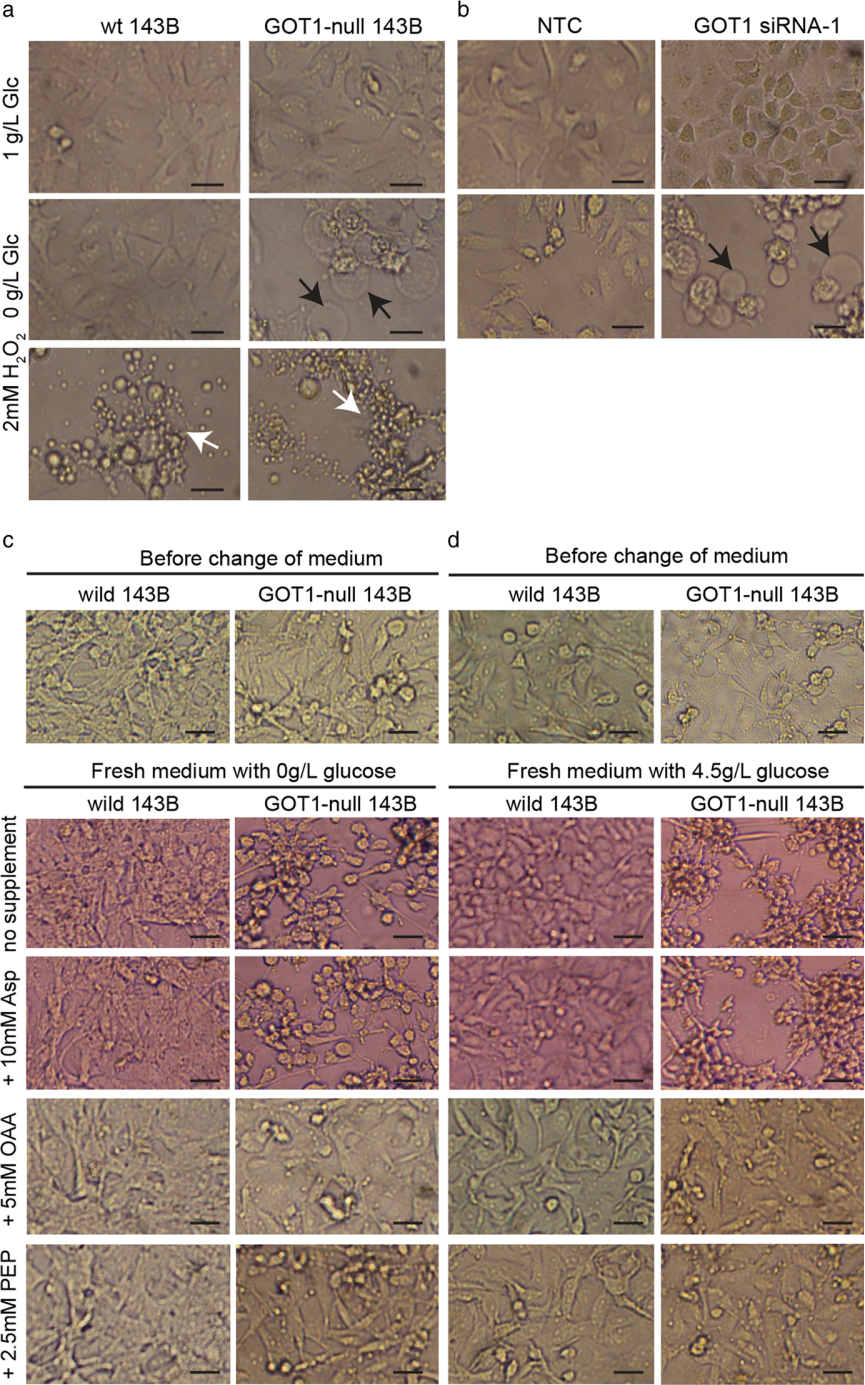
Cell death and morphology in GOT1-null 143B and siRNA knock-down A549 cells upon glucose deprivation. **a** Cells grown in 1 g/L glucose, 0 g/L glucose or treated with 2 mM H_2_O_2._for 24 h Arrows in black indicate ischemic-like cell death in GOT1-null 143B cells. Arrows in white indicate apoptotic cell death. Bars represent 25 μm. **b** A549 non-template control (NTC) and GOT1-siRNA A549 cells grown in 1 g/L glucose or 0 g/L glucose for 24 h. Arrows in black indicate ischemic-like cell death. Bars represent 25 μm. **c** Prevention of ischemic-like cell morphological changes by OAA and PEP in medium without glucose. Cells grown in 4.5 g/L glucose for 4 days, and then the old medium was replaced with glucose-free-medium containing 10 Mm Asp, 5 mM OAA or 2.5 mM PEP. The cell morphological changes 5 min after replacing nutrient-depleted-medium. **d** Prevention of ischemic-like cell morphological changes by OAA and PEP in medium with 4.5 g/L glucose. Cells grown in 4.5 g/L glucose for 4 days, and then the old medium was replaced with 4.5 g/L glucose-medium containing 10 Mm Asp, 5 mM OAA or 2.5 mM PEP. The cell morphological changes 5 min after replacing nutrient-depleted-medium. Bars indicate 25 μm


The GOT1-null cells died in a pattern similar to ischemic-like cell death upon glucose deprivation. GOT1-null cells clumped together with large and clear blisters when deprived of glucose, in contrast to the typical fragmentation into small apoptotic bodies when treated with H_2_O_2_ (Fig. 5a). Such pattern of morphological changes was also found in the GOT1 knock-down A549 cells (Fig. 5b). In addition, the replacement of the nutrient-depleted-medium with fresh medium with or without glucose triggered immediate cell morphological changes with cells pulling apart and swelling within 5 min (Additional file 4: Movie S1 and Additional file 5: Movie S2). In place of glucose, only supplementation with OAA or PEP was able to prevent the occurrence of this cell death pattern (Fig. 5c and d).

**Additional file 4: Movie S1.** Time-lapse imaging of cell morphological changes after replacing nutrient-depleted medium with fresh glucose-free DMEM. (MOV 11131 kb)



**Additional file 5: Movie S2.** Time-lapse imaging of cell morphological changes after replacing nutrient-depleted medium with fresh DMEM with glucose concentration at 4.5 g/L. (MOV 13309 kb)


### GOT1 levels in different cancer types and correlation to overall survival rate

**Fig. 6:**
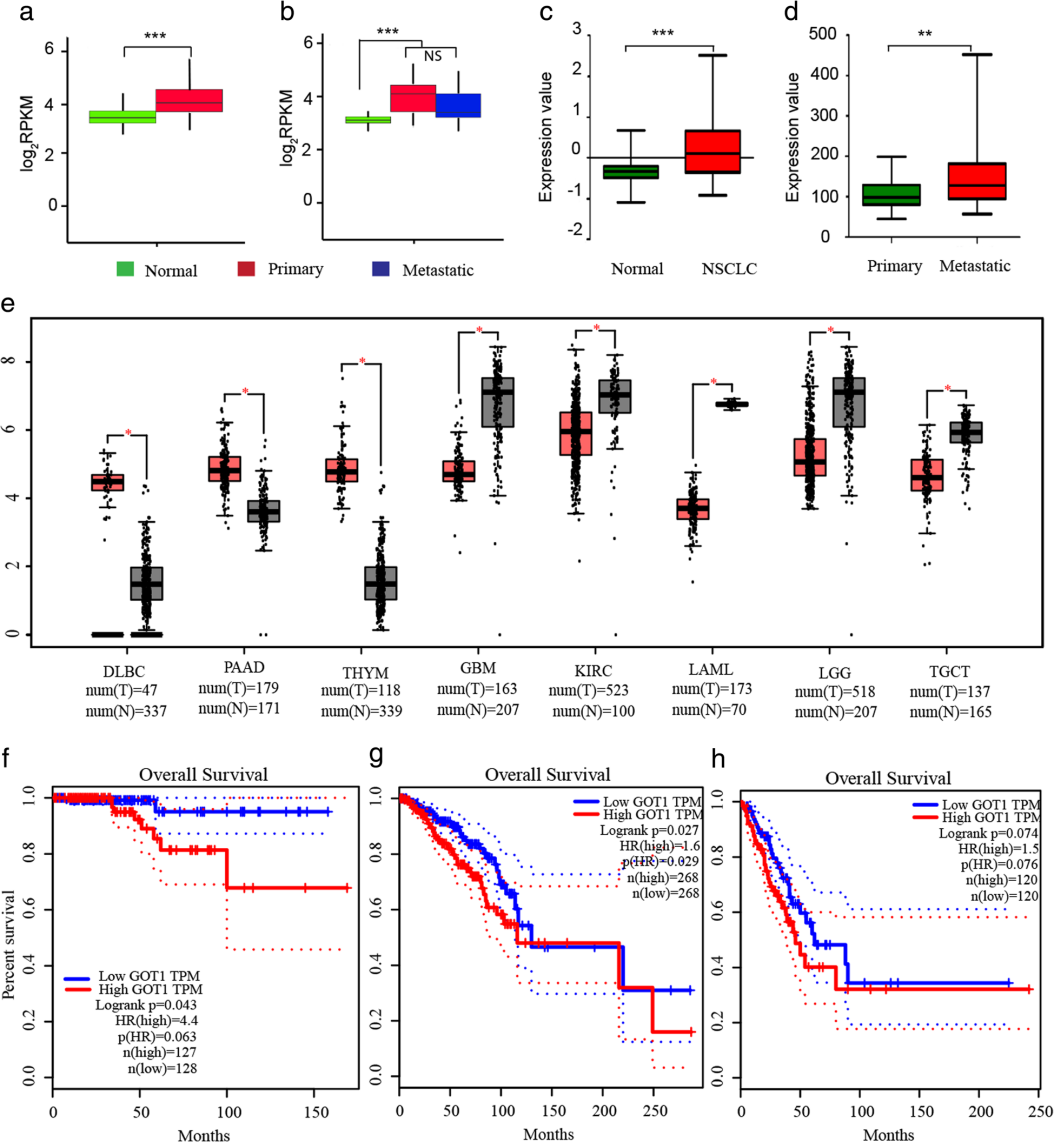
GOT1 expression in different types of cancer and the involvement in overall survival rate. **a** GOT1 mRNA expression in normal control tissue (*n* = 56) and primary lung adenocarcinoma tissue (n = 56). Wilcoxon test was performed. **b** GOT1 mRNA expression in normal control tissue (*n* = 26), primary lung adenocarcinoma tissue (n = 26) and metastatic adenocarcinoma (n = 26). All 26 subjects contributed with normal, primary and metastatic samples. Wilcoxon test was performed. **c** GOT1 mRNA expression in normal control samples (*n* = 53) and early Non-small cell lung carcinoma samples (NSCLC) (*n* = 92) analyzed from the GEO:GSE database. Unpaired student’s t-test was performed. **d** GOT1 mRNA expression in primary melanoma (*n* = 30) and metastatic melanoma (n = 53) analyzed from the GEO:GSE database. Unpaired student’s t-test was performed. **e** The diversity of expression of GOT1 in different types of cancers analyzed by the GEPIA web tool. DLBC: lymphoid neoplasm diffuse large B-cell lymphoma; PAAD: pancreatic adenocarcinoma; THYM: thymoma; GBM: glioblastoma multiforme; KIRC: kidney renal clear cell carcinoma; LAML: acute myeloid leukemia; LGG: brain lower grade glioma; TGCT: testicular germ cell cancers; **f**-**h** The relationship of GOT1 mRNA expression and overall survival rate analyzed by the GEPIA web tool, in **f**, thyroid carcinoma, **g**, in breast invasive carcinoma and **h**, in lung adenocarcinoma. *** *p* < 0.001; ** *p* < 0.01; * *p* < 0.05; NS: not significant

The GOT1 mRNA level analyzed in 56 primary lung adenocarcinoma samples was significantly higher than that in normal control tissue (Fig. 6a). However, the difference between primary and metastatic cancer tissue was not significant (Fig. 6b). Consistent with the data from our tissue data set, we found that GOT1 expression levels were up-regulated in another Non-small cell lung carcinoma data set (Fig. 6c). Interestingly, the GOT1 level was significantly higher in metastatic melanoma than primary melanoma tissues (Fig. 6d). The GEPIA web tool was used to examine the GOT1 gene expression in 33 types of tumors and it showed a diversity of expression of GOT1 in the different types of cancer (Fig. 6e). The GOT1 expression levels were significantly increased in lymphoid neoplasm diffuse large B-cell lymphoma (DLBC), pancreatic adenocarcinoma (PAAD) and thymoma (THYM), while the levels were significantly decreased in glioblastoma multiforme (GBM), kidney renal clear cell carcinoma (KIRC), acute myeloid leukemia (LAML), brain lower grade glioma (LGG) and testicular germ cell tumors (TGCT). The analysis of overall survival rate also indicated that a high level of GOT1 expression was linked to poor survival rate in certain types of tumors, including thyroid carcinoma, breast invasive carcinoma and lung cancer (Fig. 6 f-h).

## Discussion

The microenvironment in solid tumors is constantly changing and the nutrient levels will accordingly be affected. Adaptation to these changes is important for survival of tumor cells and published data have demonstrated that GOT1 plays a key role in the metabolic reprogramming and maintenance of redox homeostasis in cancer cells. In this study, we focused on the role of GOT1 in the metabolic adaptation to changes of nutrient levels. Our results show that GOT1 plays a key role to coordinate glucose consumption and glycolysis for cancer cells to survive the stress of low levels of nutrients. Inhibition of GOT1 led to glucose dependency and increased vulnerability to low nutrient levels. We found that knockout of GOT1 had neglectable effect on cell growth and survival when nutrients are sufficient. However, the GOT1-null or down-regulated cancer cells were highly sensitive to nutrient depletion or glucose deprivation. Withdrawal of glucose led to rapid death of both GOT1-null 143B and GOT1 siRNA knockdown A549 cancer cells compared to their controls. Interestingly, only OAA and PEP were able to partially rescue the cells. Although cancer cells with defects in electron transport chain were able to produce aspartate from OAA to support proliferation [5], addition of aspartate could not rescue either the GOT1-null or GOT1 siRNA knockdown A549 cancer cells. Since OAA can be converted to PEP by PEPCK, these results indicate that OAA is a key intermediate in rapidly altered metabolism in cancer cells exposed to unfavorable growth conditions. In addition to GOT1, OAA can, in mammalian cells, be produced by malate dehydrogenase, via conversion of malate to OAA, and by ATP citrate lyase, via conversion of citrate to OAA. Supplement of malate and succinate to GOT1-null cells could not rescue the cells indicating that malate dehydrogenases and ATP citrate lyases are not major sources of OAA when glucose levels are decreased. Our data suggest that GOT1 is the main source of OAA necessary for cancer cells to maintain cell viability upon nutrient scarcity.

Both OAA and PEP are substrates for gluconeogenesis. Although gluconeogenesis has been thought to exclusively occur in certain organs, PEPCK, the mitochondrial isoform of PEPCK and fructose-1,6-bisphophatase, are key enzymes in the gluconeogenesis pathway that have been shown to be involved in cancer cell proliferation and inhibition of those enzymes significantly inhibited cancer growth [30–32]. Recently, it was shown that carbons from ^13^C3-lactate appeared in PEP along the gluconeogenesis pathway during glucose deprivation in A549 and H23 lung cancer cell lines [30–33]. In our study the gene expression of *G6PC3,* an enzyme catalyzing the last step in the gluconeogenic and glyconeolytic pathways, was significantly increased in GOT1-null 143B cells. In addition the expression of BIP, a glucose-regulated protein, was also changed. Our study suggests that GOT1 is important for intracellular glucose homeostasis. Supplementation with substrates up-stream of GOT1 in the gluconeogenesis pathway did not improve cell viability in GOT1-null cells grown in glucose free medium, further supporting a pivotal role of GOT1 in providing metabolites necessary for gluconeogenesis. Galactose supports cancer cell proliferation mainly through fueling the pentose phosphate pathway and not glycolysis [34]. Therefore, the slightly increased viability by addition of galactose indicated that the pentose phosphate pathway partially contributed to cell viability, but was not the key pathway for GOT1-null cells to survive glucose deprivation. Metformin is a well-known gluconeogenesis inhibitor that has been shown to cause accumulation of NADH in cells [31]. A similar pattern of NADH accumulation was found in GOT1-null 143B cells grown in nutrient-depleted conditions. Supplement with NAD^+^ improved the NADH/NAD^+^ ratio and could partially rescue the GOT1-null cells grown in nutrient-scarcity.

Pyruvate conversion to lactate is one of the major sources to regenerate NAD^+^ in cells [35]. In GOT1-null 143B cells, the lactate secretion rate was considerably higher than in wild type control cells. This data indicated that increased glucose consumption might be utilized to support the pyruvate-to-lactate-reaction and thereby regenerate NAD^+^ to balance up the accumulation of NADH in GOT1-null cells. The addition of pyruvate was able to protect GOT1-null 143B to maintain viability upon glucose withdrawal. The recently published work by Abrego showed that up-regulation of GOT1 decreased the lactate release rate in low pH microenvironment [36]. Instead of making macromolecules for cell proliferation, pyruvate was secreted into the extracellular microenvironment. Such non-economic metabolism occurred at the expense of glucose dependency. Actually, the GOT1-null 143B cells increased autophagy, as a possible compensatory mechanism. When nutrient levels were high, the new rewired pathways could function well. However, when the nutrient levels dropped, the GOT1-null cells had lost the metabolic flexibility to survive unfavorable conditions [25, 26]. Furthermore, inhibition of GOT1 led to rapid ischemic-like cell death upon glucose deprivation and only OAA and PEP were able to fully prevent, and NAD^+^ partially prevent such morphological changes. The ischemic-like cell death is also called oncosis and has been shown to be involved in diseases such as ischemic heart disease and stroke [37–39]. Consistent with our results, other studies have shown that OAA is neuroprotective against ischemic stroke and that a combination of human rGOT1 with low doses of OAA induces a protective effect after cerebral ischemia [40, 41]. Moreover, evidence also indicates that NAD^+^ is able to protect cells against ischemic injury [42, 43].

Finally, analysis of a sequencing dataset and mRNA expression datasets showed that increased GOT1 expression is found in lung adenocarcinoma and melanoma. Moreover, higher levels of GOT1 were linked to poor survival in certain types of cancers. However, we also found GOT1 significantly decreased in other types of cancers. The diversity of GOT1 expression is probably a result of the pivotal role of GOT1 in cancer metabolic plasticity. Cancer cells are able to control the aerobic glycolysis rate via GOT1 and thereby regulate the NADH/NAD^+^ ratio. Up- or down-regulation of GOT1 most likely depends on cancer type, growth properties and micro environment. GOT1 might be a useful candidate target for treatment of cancers with high GOT1 expression.

## Conclusions

In summary, our study shows that GOT1 plays a key role in rapid metabolic adaptation to nutrient level changes through coordinating the glucose consumption and the glycolysis rate. Further experiments are needed to elucidate how GOT1 can be targeted in treatment of cancers expressing high levels of GOT1.

## Additional files

Additional file 1: **Figure S1.** Characterization of GOT1 siRNA-inhibited 143B and A549 cells. a, Establishment of GOT1 siRNA knock-down in 143B cells. Mean ± s.d. from 3 independent experiments. b, Growth curve of siRNA-1 143B cells. Mean ± s.d. from representative one of 3 independent experiments. c, Colony formation of siRNA-1 143B cells. Mean ± s.d. from representative one of 3 independent experiments. d, Migration of siRNA-1 143B cells. Bars indicate 50 μm. e, Establishment of siRNA knock-down A549 cells. Mean ± s.d. from 3 independent experiments. f, Colony formation of siRNA-1 A549 cells. Mean ± s.d. from representative one of 3 independent experiments. Unpaired student’s t-test was performed. *** *p* < 0.001; ** *p* < 0.01; *p < 0.05. (TIF 1048 kb)

Additional file 2: **Figure S2.** Rescue of GOT1 down-regulated and GOT1-null cells by oxaloacetate. Relative cell viabilities after 8 h (a) and 24 h (b) in wild type 143B cells and 8 h (c) and 24 h (d) in wild type A549 cells. Relative cell viabilities after 8 h (e) and 24 h (f) in GOT1 siRNA knock-down A549 cells. Rescue of GOT1-null 143B cells with OAA at different concentrations upon glucose deprivation (g). Mean ± s.d. from 3 independent experiments. One-way ANOVA test was performed. *** *p* < 0.001; ** *p* < 0.01;* *p* < 0.05. NS: not significant. (TIF 230 kb)

Additional file 3: **Figure S3.** Partial prevention of ischemic-like-cell-death morphological changes by NAD^+^. Bars indicate 25 μm. (TIF 1353 kb)

## Abbreviations

AOA: Aminooxyacetate
DLBC: Lymphoid neoplasm diffuse large B-cell lymphoma
G6PC3: Glucose-6-phospholase
GBM: Glioblastoma multiforme
GOT1: Glutamate Oxaloacetate Transaminase 1
H_2_O_2_: Hydrogen peroxide
KIRC: Kidney renal clear cell carcinoma
LAML: Acute myeloid leukemia
LGG: Brain lower grade glioma
OAA: Oxaloacetate
PAAD: Pancreatic adenocarcinoma
PEP: Phosphoenolpyruvate
TGCT: Testicular germ cell tumors
THYM: Thymoma
