# Supplementary figures and images for: Inhibition of glutamate oxaloacetate transaminase 1 in cancer cell lines results in altered metabolism with increased dependency of glucose
Source: BMC Cancer. 2018 May 11;18:559. doi: 10.1186/s12885-018-4443-1 (PMC5948873; doi:10.1186/s12885-018-4443-1)

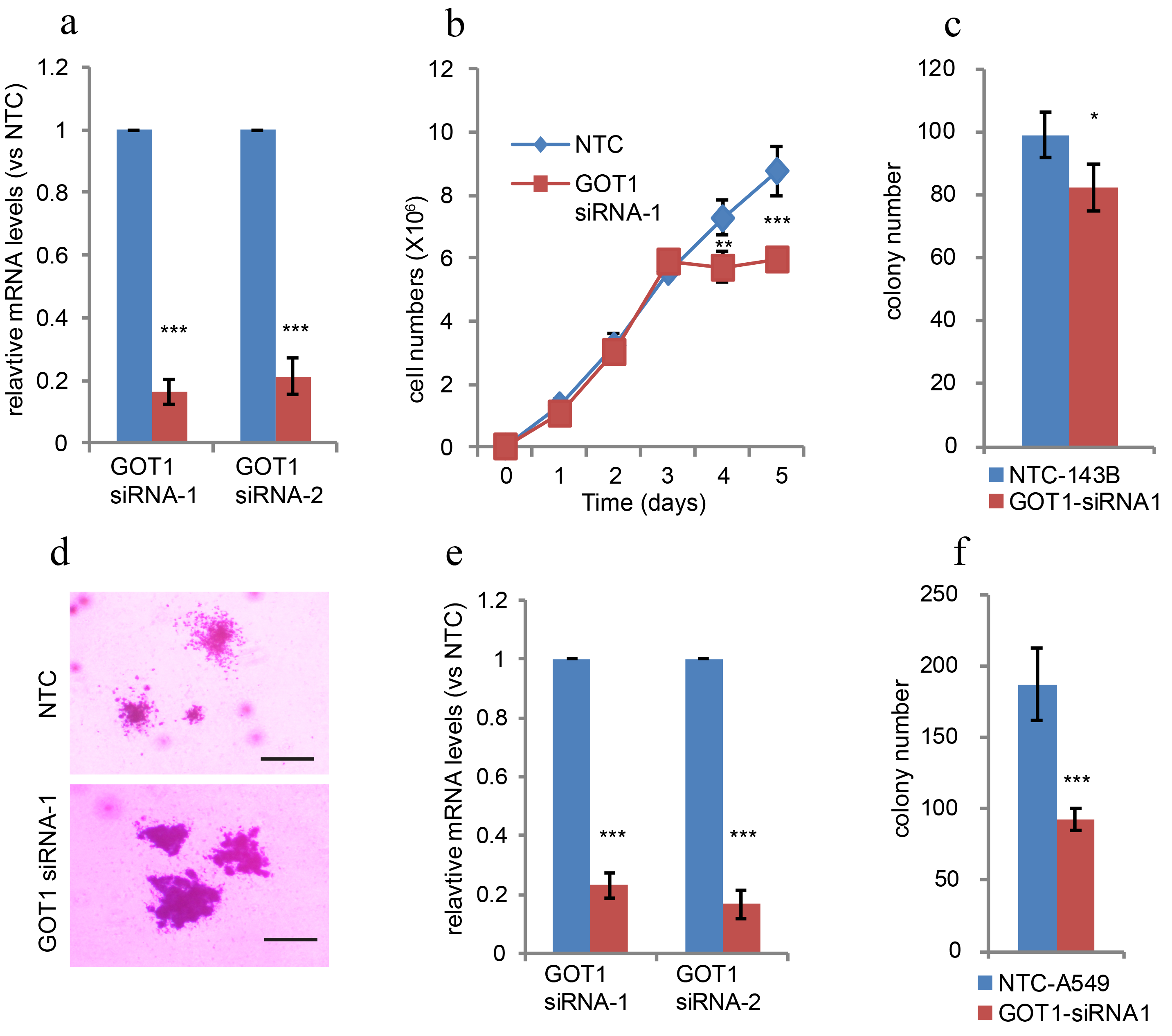

Supplement: Supplementary file 3 — Figure S1. Characterization of GOT1 siRNA-inhibited 143B and A549 cells. a, Establishment of GOT1 siRNA knock-down in 143B cells. Mean ± s.d. from 3 independent experiments. b, Growth curve of siRNA-1 143B cells. Mean ± s.d. from representative one of 3 independent experiments. c, Colony formation of siRNA-1 143B cells. Mean ± s.d. from representative one of 3 independent experiments. d, Migration of siRNA-1 143B cells. Bars indicate 50 μm. e, Establishment of siRNA knock-down A549 cells. Mean ± s.d. from 3 independent experiments. f, Colony formation of siRNA-1 A549 cells. Mean ± s.d. from representative one of 3 independent experiments. Unpaired student’s t-test was performed. *** p < 0.001; ** p < 0.01; *p < 0.05. (TIF 1048 kb) [file 12885_2018_4443_MOESM1_ESM.tif]

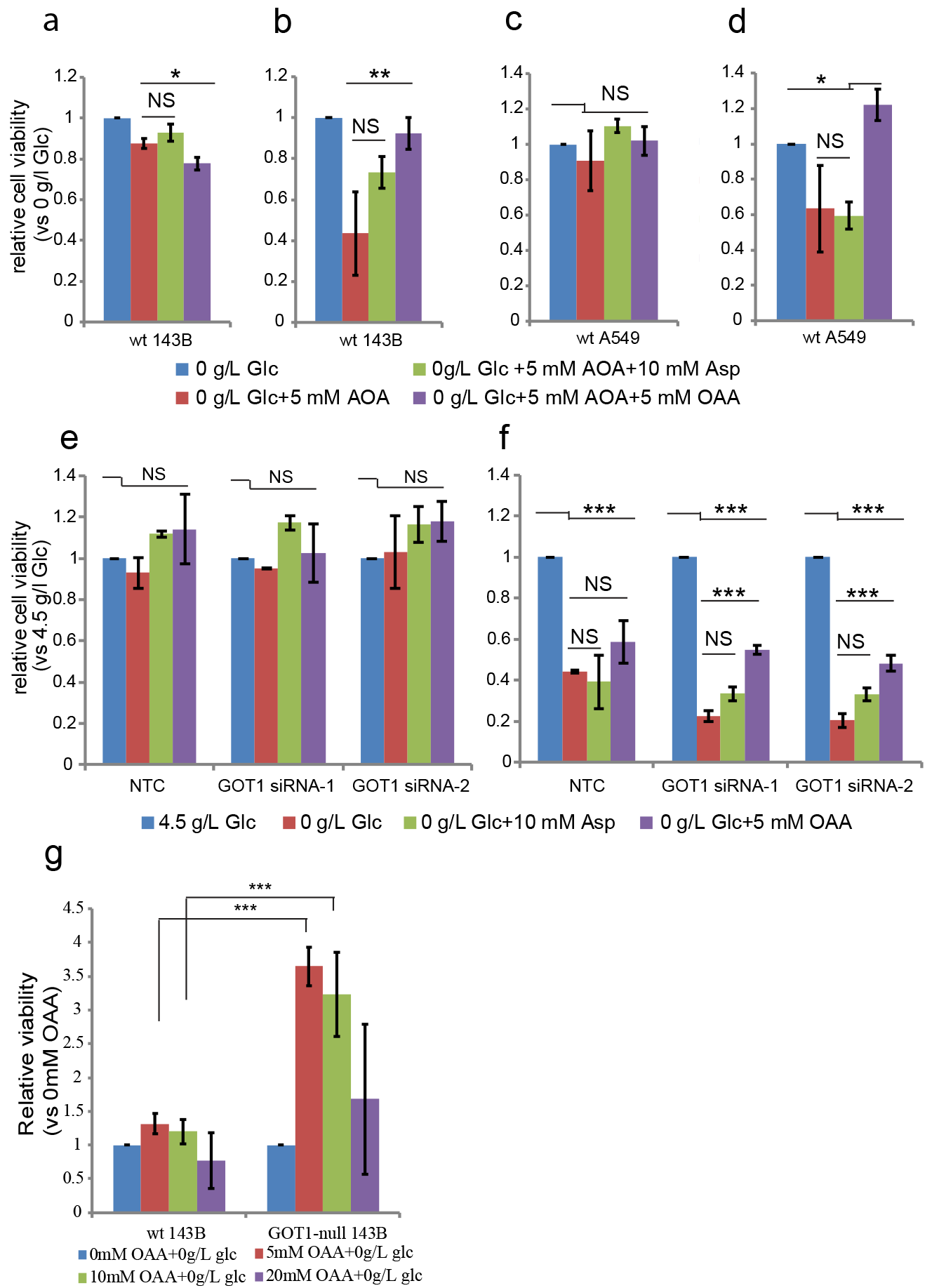

Supplement: Supplementary file 4 — Figure S2. Rescue of GOT1 down-regulated and GOT1-null cells by oxaloacetate. Relative cell viabilities after 8 h (a) and 24 h (b) in wild type 143B cells and 8 h (c) and 24 h (d) in wild type A549 cells. Relative cell viabilities after 8 h (e) and 24 h (f) in GOT1 siRNA knock-down A549 cells. Rescue of GOT1-null 143B cells with OAA at different concentrations upon glucose deprivation (g). Mean ± s.d. from 3 independent experiments. One-way ANOVA test was performed. *** p < 0.001; ** p < 0.01;* p < 0.05. NS: not significant. (TIF 230 kb) [file 12885_2018_4443_MOESM2_ESM.tif]

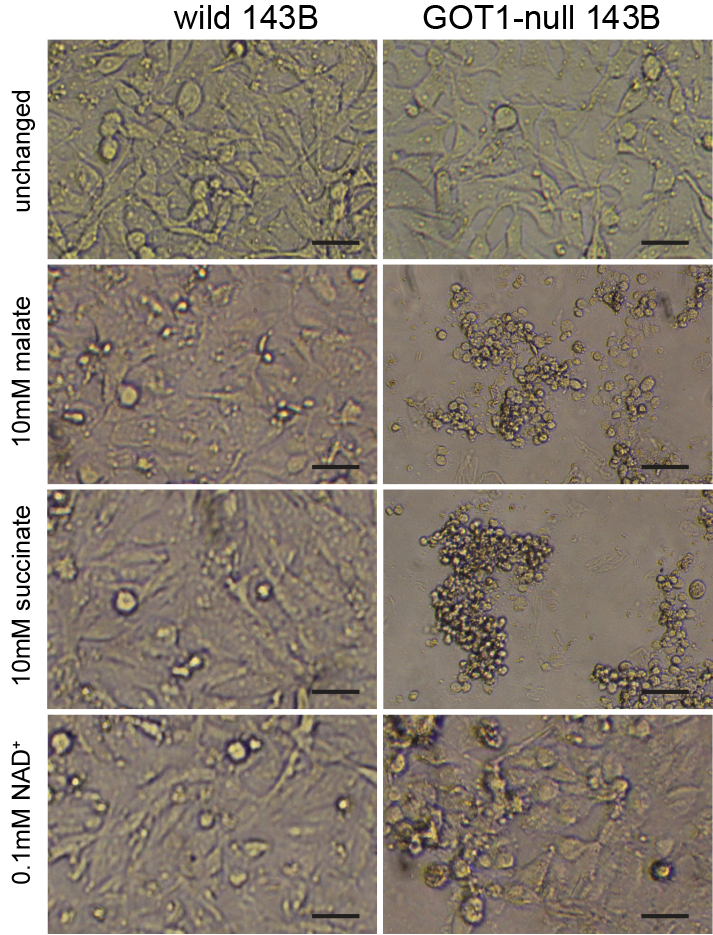

Supplement: Supplementary file 5 — Figure S3. Partial prevention of ischemic-like-cell-death morphological changes by NAD+. Bars indicate 25 μm. (TIF 1353 kb) [file 12885_2018_4443_MOESM3_ESM.tif]
